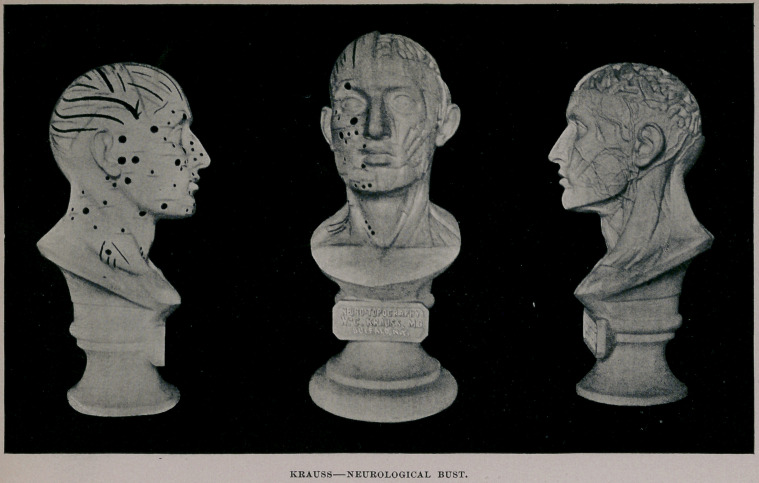# A Neurological Bust1Read before the Central New York Medical Association, Buffalo, N. Y., October 16, 1894.

**Published:** 1895-03

**Authors:** William C. Krauss

**Affiliations:** Buffalo, N. Y.


### A NEUROLOGICAL BUST.[1]

By WILLIAM C. KRAUSS, M. D., Buffalo, N. Y.

At the seventeenth annual meeting of the American Neurological Association, held at Washington, D. C., September 22-24, 1891, I exhibited a bust,[2] which was designed particularly for the neurologist, as a means of a ready reference in his consultation room, and for the teacher in neurology, as an important aid in the elucidation of facts in class-room demonstration. The features to which attention was called were the fissures of the brain repre-

1. Read before the Central New York Medical Association, Buffalo, N. Y., October 16, 1894.
2. *Journal of Nervous and Mental Disease*, December, 1891.

KRAUSS—NEUROLOGICAL BUST.

sented by grooved lines on the head and the electro-motor points of the muscles and nerves of the face, represented by grooved circles. Both sides of the bust were exactly alike.

I have recently improved the bust in several ways and believe its value and efficiency to be greatly enhanced thereby.

The right side shows the relative position of the fissures and convolutions of the brain to the sutures and bones of the skull, the fissures being represented by grooved lines, deep or shallow, according to the size of the respective fissures in the brain. To render them more distinct and visible they have been traced in black. The principal sutures of the cranium are represented by zig-zag lines traced in blue.

The face and neck present the electro-motor points of the muscles and nerves, the muscles being colored red, the nerves yellow.

The left side of the bust reveals the underlying anatomical structure and herein does the new bust differ materially from the old. The skull cap has been removed sufficiently to disclose the left hemisphere of the brain *in situ*, with its fissures and convolutions fairly accurately portrayed.

The dissection of the muscles of the face and neck, the arteries, veins and a few nerves has been skilfully executed and is the work of an experienced anatomical sculptor, aided by the advice of prominent anatomists, insuring the greatest amount of accuracy compatible with work of this kind.

Aside from the scientific worth and importance, the artistic beauty of the bust, as revealed in the plate, will be generally recognized.

### CRANIAL REGION.

*Fissures.*—Sylvian fissure, central fissure (Rolando), parietal fissure, occipital fissure, superior frontal convolution (superfrontal), middle frontal convolution (medi-frontal), inferior frontal convolution (sub-frontal), ascending frontal convolution (precentral), ascending parietal convolution (postcentral), superior parietal convolution (parietal), inferior parietal convolution (subparietal), angular convolution, superior temporal convolution (supertemporal), middle temporal convolution (meditemporal), inferior temporal convolution (subtemporal), superior occipital convolution, middle occipital convolution and inferior occipital convolution.

*Sutures.*—Coronal suture, squamous suture, lambdoid suture and sagittal suture.

FACIAL REGION.

*Nerves.*—Trifacial nerve, superior branch; trifacial nerve, middle branch; trifacial nerve, trunk; trifacial nerve, inferior branch; hypoglossal nerve, accessorius nerve, Erb's point (supraclavicular point), phrenic nerve, brachial plexus, axillary nerve, infraorbital nerve, supraorbital nerve.

*Muscles.*—Frontalis, corrugator supercilii, orbicularis palpebrarum, nasal muscles, zygomatic muscles, orbicularis oris, masseter, levator menti, quadratus menti (depressor labii inferioris), platysma myoides, hyoid muscles, sterno-cleido-mastoid, omohyoid, splenicus, trapezius, levator anguli scapuli, triangularis menti (depressor anguli oris), stylo-hyoid, digastric, risorius and levator labii superioris.

## Figures and Tables

**Figure f1:**